# Thirty-Day Surgical Morbidity and Mortality in Pelvic and Acetabular Fracture Patients Presenting to a Tertiary Care Hospital in Karachi, Pakistan

**DOI:** 10.7759/cureus.63801

**Published:** 2024-07-04

**Authors:** Hammar Shahid, Masood Umer, Marij Zahid

**Affiliations:** 1 Department of Orthopaedic Surgery, Aga Khan University Hospital, Karachi, PAK

**Keywords:** trauma management, morbidity after pelvic fracture fixation, pelvic fracture fixation, poly trauma, acetabulum fracture fixation, acetabulum fracture, pelvic fracture

## Abstract

Introduction: Pelvic fractures, encompassing a spectrum from minor to life-threatening injuries, pose challenges in trauma management. This study focuses on short-term outcomes, exploring morbidity and mortality within 30 days postoperative, among pelvic fracture patients at a tertiary care hospital in Karachi, Pakistan. The majority of pelvic injuries result from intense blunt trauma, with associated risks of concomitant injuries. Pelvic fractures are linked to early complications such as hemorrhage, thromboembolism, and infections, influencing mortality rates.

Methodology: A prospective cohort study involving 53 surgically managed pelvic fracture patients was conducted at Aga Khan University Hospital, Karachi. Variables such as age, gender, comorbidities, mechanism of injury, associated injuries, and presenting vitals were documented. Thirty-day morbidity included surgical site infections, hemorrhagic shock, nerve injuries, and others. Statistical analyses assessed associations between patient characteristics and morbidity.

Results: The study revealed a median age of 37 years, with 77% male patients. Most fractures result from motor vehicle accidents. Morbidity occurred in 31.6% of cases, primarily associated with the presence of associated injuries. Postoperative complications included neurological deficits (15.1%) and pulmonary complications (9.4%). No 30-day mortality was reported.

Discussion: The study highlights the importance of a multidisciplinary approach in managing pelvic fractures, emphasizing the association between associated injuries and postoperative morbidity. Comorbidities did not significantly impact morbidity, emphasizing the traumatic nature's independent contribution. Timely presentation (median 20 hours) and efficient trauma systems are crucial for optimal outcomes.

Conclusion: This research contributes insights into short-term outcomes following pelvic fracture fixation in a Pakistani tertiary care setting. By exploring a range of parameters, the study emphasizes the need for comprehensive management strategies to minimize complications and improve patient outcomes. Bridging critical knowledge gaps, this research informs clinical decision-making for pelvic fracture patients in this region.

## Introduction

Pelvic injuries span a spectrum from minor to potentially life-threatening. These encompass fractures of the pelvic ring, acetabular fractures, and avulsion injuries. The majority of pelvic injuries in younger people are caused by intense blunt trauma, although elderly people may sustain these injuries from lower-energy sources including falling from a standing posture. Patients with high-energy trauma have an increased risk of concomitant injuries, especially involving the abdominal and pelvic organs. Pelvic ring injuries have an incidence of 20-37/100,000 in the general population [1-3]. Pelvic fractures can be categorized as stable or unstable injuries and as open or closed fractures (based on the presence or absence of a wound communicating with fracture hematoma). The overall mortality in isolated pelvic ring injuries has been reported 5% and up to 35% and 46% in poly-trauma patients [4-6]. Unstable and open pelvic fractures have been associated with an increased risk of mortality and morbidity of 45% [7,8]. Hemorrhage, thromboembolism, infection, associated genitourinary, soft tissue de-gloving injuries, and gastroenterological and neurological injuries are all early complications [8,9]. Surgical site infections, screw malposition, hemorrhagic shock, bladder injury, vascular injury, and implant failure have all been reported secondary to fixation [10].

Understanding the pelvic ring and acetabulum anatomy is of utmost importance as it is complex. Occult fractures of the pelvis and acetabulum are easily missed on plain radiographs, and hence the modality of advanced imaging comes into play. CT scan with 3D reconstruction is the gold standard nowadays for the diagnosis of these complex injuries. Multiple classification systems have been used, most commonly being Young and Burge’s and AO's classification systems for pelvic ring injuries, while Judet and Letournel's classification is still utilized for acetabulum fractures. Management of these injuries commences with prompt response in the emergency room, adequate resuscitation including blood transfusions, use of pelvic binders for temporary stabilization, or even an external fixator [11]. Identification of associated injuries and utilizing a multi-disciplinary approach is of utmost importance. Gustavo et al. reported that outcomes of pelvic fracture have been associated with severity of associated injuries and not the fracture type [12]; however, various studies document that mortality has been associated with fracture pattern [13,14].

Pelvic fracture fixation is a common intervention aimed at stabilizing the pelvic ring and reducing associated morbidity and mortality. However, the comprehensive understanding of the outcomes following pelvic fracture fixation remains an area of active research. The primary rationale for our study lies in the need to assess the short-term morbidity and mortality outcomes in patients undergoing pelvic fracture fixation at a tertiary care center. By focusing on the 30-day postoperative period, we aim to capture critical events and complications that may directly result from or in combination with the surgical intervention. The specific objectives of our study included quantification of morbidity rates and mortality rates and investigation to identify any trends or factors associated with increased morbidity and mortality following pelvic fracture fixation.

Pelvic fracture fixation is a common intervention aimed at stabilizing the pelvic ring and reducing associated morbidity and mortality. However, the comprehensive understanding of the outcomes following pelvic fracture fixation remains an area of active research. The primary rationale for our study lies in the need to assess the short-term morbidity and mortality outcomes in patients undergoing pelvic fracture fixation at our tertiary care center. By focusing on the 30-day postoperative period, we aim to capture critical events and complications that may directly result from or in combination with surgical intervention. The findings from this study will contribute to the improvement of patient care by providing evidence-based insights into the short-term outcomes of pelvic fracture fixation. In conclusion, our research endeavors to fill a critical gap in the current understanding of the immediate postoperative period following pelvic fracture fixation. The outcomes of this study not only contribute to the academic knowledge base but, more importantly, have direct implications for the clinical management and outcomes of patients undergoing pelvic fracture fixation at our tertiary care center.

## Materials and methods

Approval was sought from the Ethics Review Committee, AKUH (IRB # 2022-0525-22307). This was a prospective cohort study done at the section of Orthopedics, Department of Surgery, Aga Khan University Hospital, Karachi, Pakistan. A total of 53 patients who underwent surgical fixation for pelvic ring or acetabulum fractures from January 2021 to December 2022 were included in the study. All pelvic fixations were performed by one of the three fellowship-trained pelvic surgeons. Non-probability consecutive sampling technique was employed. The data were imported from our hospital’s trauma registry after approval from the Ethical Review Committee and documented on a pre-structured proforma, including variables of interest. The variables included age, gender, comorbidities, time to presentation, mechanism of injury, associated injuries, presenting vitals, consciousness level according to the Glasgow coma scale (GCS), and classification of injury. Thirty-day morbidity was defined as an occurrence of surgical site infection, hemorrhagic shock, bladder injury, nerve injury, vascular injury, screw malposition, prolonged intensive care stay (>3 days), pulmonary embolism, respiratory tract infections, need for transfusions, cardiac events, sepsis, and implant failure in patients with pelvic fracture, who were managed surgically.

For the purpose of analyzing the impact of fracture instability on morbidity and mortality, we sub-classified fractures into type O (no instability), type R (rotational instability), or type RV (rotational and vertical instability). Pure acetabular fractures were classified as type O as they are stable. Stata (version 15.2; StataCorp LLC, College Station, TY) was used for data entry and analysis. Quantitative variables (age, presenting vitals, length of stay, number of transfusions) were expressed as mean ± standard deviation or median (IQR), and qualitative variables were calculated as frequencies and percentages. Patients were divided into two groups based on the presence or absence of morbidities (primary outcome). Comparison of continuous variables was done using a t-test, while categorical variables were compared using the chi-square test or Fischer's exact test. Univariate analysis was done using simple logistic regression, and those significant were tested using multiple logistic regression after testing for multicollinearity, keeping significance level < 5%.

## Results

A total of 53 patients who met the inclusion criteria were included in the study. The median age of the patients was 37 years (IQR: 28-50). Seventy-seven percent of patients (n=41) were males, and 22.6% (n=12) were females. Seventy-five percent (n=40) of our patients had no associated comorbidity, while 7.54% (n=4) had single comorbidity, and 16.98% (n=9) had multiple comorbidities. The median time from injury to presentation was 20 hours (IQR: 8-42) (Table 1). The mean presenting mean arterial pressure (MAP) was 91.99 ± 15.64. Grade 1 shock was seen in 60.4% (n=32) at the time of presentation, 26.4% (n=14) had grade II shock, and 13.2% (n=7) had grade III shock (Table 2). The GCS at presentation was 15 for 98.1% (n=52) of patients. The associated injuries included abdominal injuries in 22.7% (n=12), lower limb fractures in 22.7% (n=12), thoracic injuries in 20.8% (n=11), bladder injury in 20.8% (n=11), upper limb fractures in 15.1% (n=8), spine involvement in 5.7% (n=3), and traumatic brain injury/head injury in 5.7% (n=3). The majority of the patients had closed fractures, except one who had open pelvic fractures (Table 2). The most common mechanism of injury was motor vehicle accidents caused by high-velocity blunt trauma (n=44, 83%), followed by fall from height (n=8, 15%) and firearm or missile injury (n=1, 2%).

**Table 1:** Injury and peri-operative qualitative parameters *SD - standard deviation

Variable	Mean ± SD*
Time to injury (hours)	35.5 ± 37.2
Injury severity score (ISS)	11.9 ± 6.1
Blood loss (mL)	445.8 ± 484
Drop in Hb (g/dL)	1.5 ± 1.2

**Table 2:** Injury and peri-operative qualitative parameters *ICU - intensive care unit, ** PCV - packed cell volume

Variable	n (%)
Shock	
Grade I	32 (60.4)
Grade II	14 (26.4)
Grade III	7 (13.2)
Injury type	
Close	51 (98.1)
Open	1 (1.9)
ICU* stay	
None	45 (84.9)
1-2	5 (9.4)
3-4	3 (5.7)
Special care stay	
None	26 (49.1)
1-2	21 (39.6)
3-4	3 (5.7)
>4	3 (5.7)
PCV** transfusion	
None	24 (45.3)
1	3 (5.7)
2	12 (22.6)
3	4 (7.5)
>3	10 (18.9)

Among pelvic fractures classified based on the AO Foundation/Orthopaedic Trauma Association (AO/OTA) (AOOTA) classification, the most prevalent was 61c (32.1%, n=17), followed by 61b (26.4%, n=14) and then 61a (3.8%, n=2). Based on Young and Burgess's classification, LC3 was the most common injury, with an incidence of 15.1% (n=8), followed by AP3 at 13.2% (n=7); AP1, AP2, and LC1 at 9.4% (n=5) each; and VS 5.7% (n=3). The most common acetabulum fractures were 62a (26.41% n=14), followed by 62b (20.75%, n=11) and 62c (16.98%, n=9). Based on Lunsjo et al.'s classification, 28.3% (n=15) were type O, 32.1% (n=17) were type R, and 39.6% (n=21) were type RV.

The median intraoperative blood loss was 250 mL (IQR: 100-500) (Table 1). Fifteen percent of the patients (n=8) and 50.9% (n=27) required postoperative ICU and SCU stays, respectively. Out of these, 9.4% (n=5) remained in the ICU for two or fewer days, and 5.7% (n=3) had a stay of three or more days. The special care stay was two or fewer days in 39.6% (n=21) patients, three to four days in 5.7% (n=3), and more than four days in 5.7% (n=3) as well (Table 2). The mean HB drop was 1.5 ± 1.20 mg/dL. Forty-five percent (n=24) of our patients did not require transfusion, 28.3% (n=15) required 2 or fewer units of packed cells, and 26.4% (n=14) required more than two packed cells of transfusions (Table 2).

The total number of patients who developed any complication after surgical fixation was 21 (31.62%). The complications reported were postoperative neurological deficit in 15.1% (n=8), which included altered sensation on the foot, foot drop, bilateral foot drop, big toe numbness and sole numbness, and pulmonary complications in 9.4% (n=5), which included pulmonary embolism (PE) in 3.8% (n=3.8) and pneumonia in 5.7% (n=3) patients, deep vein thrombosis (DVT) in 5.7% (n=3), and surgical site infections and postoperative shock in 3.8% (n=2). PE was managed with anticoagulants in both patients with one of them requiring an inferior vena cava (IVC) filter as well, and pneumonia was treated with appropriate antibiotics. There was a 1.9% (n=1) incidence of deep infection, sepsis, small bowel obstruction, and implant failure. There were no vascular or cardiac complications or screw malposition reported in our study. As the individual number of complications was less in number, we collectively divided the patients into two groups (i.e., with or without postoperative complications/morbidity; Tables 3, 4).

**Table 3:** Association of complications with quantitative parameters *SD - standard deviation; ** ISS - injury severity score

	Complications (Mean ± SD*)		p-value
	Yes	No	
Age (years)	44.7 ± 14.1	39.9 ± 18.2	0.71
Time to presentation (hrs)	47.1 ± 35.3	31.9 ± 37.6	0.26
Mean ISS**	12.52 ± 7.45	10.03 ± 6.4	0.89
Blood loss (mL)	607.1 ± 572	388.8 ± 444.2	0.57

**Table 4:** Association of complications with qualitative parameters *ICU - intensive care unit, **ORIF - open reduction internal fixation, ***SI - sacro-iliac, ****THR - total hip replacement

	Complications n (%)		p-value
	Yes	No	
Demographics			
Male	17 (41.5)	24 (58.5)	0.61
Female	4 (33.3)	8 (66.7)	
Comorbid status			
None	15 (39.5)	23 (60.5)	0.81
Single	3 (50)	3 (50)	
Multiple	3 (33.3)	6 (66.7)	
Associated injuries			
Present	15 (55.6)	12 (44.4)	0.02
Absent	6 (23.1)	20 (76.9)	
Severity of shock			
Grade I	10 (31.3)	22 (68.8)	0.14
Grade II	6 (42.9)	8 (57.1)	
Grade III	5 (71.4)	2 (28.6	
Fracture type			
Open	0	1 (100)	0.61
Close	20 (39.2)	31 (60.8)	
Fracture classification			
Type O	4 (26.7)	11 (73.3)	0.44
Type R	7 (41.2	10 (58.8)	
Type RV	10 (47.6)	11 (52.4)	
ICU* stay (days)			
None	16 (35.6)	29 (64.4)	0.35
1-2	3 (60)	2 (40)	
>2	2 (66.7)	1 (33.3)	
Special care stay (days)			
None	6 (23.1)	20 (76.9)	0.1
1-2	11 (52.4)	10 (47.6)	
3-4	2 (66.7)	1 (33.3)	
>4	2 (66.7)	1 (33.3)	
Packed cells transfusion			
None	6 (25)	18 (75)	0.32
1	1 (33.3)	2 (66.7)	
2	6 (50)	6 (50)	
3	2 (50)	2 (50)	
>3	6 (60)	4 (40)	
Procedure			
ORIF** acetabulum	6 (25)	18 (75)	0.17
SI*** screw	5 (38)	8 (62)	
SI*** screw + ORIF** pubic symphysis	3 (60)	2 (20)	
ORIF** pubic symphysis	2 (66.7)	1 (33.3)	
ORIF** Acetabulum + ORIF** pubic symphysis + SI*** screw	3 (100)	0	
ORIF** Acetabulum + SI*** screw	1 (50)	1 (50)	
ORIF** iliac blade	0	1 (100)	
ORIF** acetabulum + THR****	1 (100)	0	
ORIF** Acetabulum + SI*** screw + ORIF** Iliac blade + ORIF** pubic symphysis	0	1 (100)	

The presence of associated injuries had a significant association with postoperative morbidity in our study (p=0.02). Age, time to presentation in hours, gender, comorbidities, severity of shock, fracture type and classification on either classification systems, intraoperative blood loss, ICU stay, SCU stay, or transfusions had no significant association with the presence or absence of complications. Fracture type had a causative trend toward complications but was not statistically significant (p=0.61) (Tables 3-4).

Univariate analysis was done to compare the two groups against the predictor variables of interest using simple logistic regression analysis. The odds of associated injuries among patients with complications was 4.16 times as compared to patients without complications (OR=1.3-13.6, p=0.01). The odds of having type R fractures (baseline Type O) amongst patients who had complications was 1.9 times more as compared to patients who did not have complications (OR=0.43-8.6), while the odds of having type RV fractures was 2.5 times more (CI=0.6-10.4). Moreover, there was a significant association of morbidity with the length of specialized care in either the ICU and/or high dependency unit (HDU). The odds of one to two days in ICU and/or HDU stay was 1.3 times (CI=0.5-2.6), and the odds of three or more days stay in ICU and/or HDU was 1.9 times (CI=0.3=3.8) among patients who had complications as compared to those who did not have any morbidity (baseline=regular ward stay). Age, gender, transfusion requirement, and blood loss had no significant association with complications. Multivariable analysis was attempted but was not significant. No interactions were observed as well among the variables.

## Discussion

Pelvic fractures, ranging from minor to life-threatening, pose significant challenges due to their association with high morbidity and mortality rates. The diverse nature of these injuries, stemming from both high- and low-energy traumas, underscores the importance of comprehensive understanding and effective management. The complexity of pelvic anatomy necessitates advanced imaging techniques such as CT scans for accurate diagnosis, given that occult fractures may be missed on plain radiographs. The prompt identification of associated injuries and a multidisciplinary approach become pivotal in optimizing patient outcomes.

Our study delves into the intricate landscape of pelvic fractures, offering a comprehensive examination of demographic factors, intraoperative parameters, and postoperative complications among 53 patients. The median age of 37 years aligns with existing literature, highlighting the prevalence of pelvic fractures across a broad age spectrum [7,15]. The majority of our patients were males, consistent with the higher incidence of pelvic fractures observed in males due to increased engagement in high-risk trauma. The morbidity and mortality rates associated with pelvic fractures are influenced by various factors, including the mechanism of injury, the presence of associated injuries, and fracture type. In our study, the overall morbidity was 31% (n=21), which aligns with existing literature, highlighting the gravity of such traumas, especially in polytrauma scenarios. Among the complications, 48% occurred in fracture-type RV patients, followed by 41% in fracture-type R0. Surprisingly, we did not encounter any 30-day mortality. This can be due to the fact that we included only those patients who underwent surgical fixation, received postop care, and were discharged, while those who did not go for surgery or might be too sick were not included.

The complications reported in our study, including postoperative neurological deficits, pulmonary complications, and deep vein thrombosis, provide a snapshot of the challenges encountered in the immediate postoperative period. Importantly, the association between the presence of associated injuries and postoperative morbidity emphasizes the need for a holistic approach to managing pelvic fractures.

The significant finding that 75% of patients had no associated comorbidities underscores the impact of trauma itself as a major determinant of outcomes in pelvic fractures. While comorbidities often exacerbate complications in orthopedic cases, our study indicates that comorbid had no impact on morbidity. Hence, the traumatic nature of pelvic fractures can independently contribute to morbidity. This is contrary to the work of Abdelrahman et al. who emphasize the need for tailored approaches in trauma patients with varying comorbidities [7].

Gustavo et al. [12] associated outcomes with the severity of associated injuries. We also found that the presence of associated injuries potentiates the early complications by 4.2 times. However, the injury severity score did not predict early postoperative morbidity, as depicted by few studies [16-18], although it contributes significantly to mortality. This focus on the 30-day postoperative period enables us to capture critical events directly linked to the surgical procedures, providing valuable insights for immediate patient management.

The median time from injury to presentation (20 hours) reflects the complexity of managing pelvic fractures, considering the need for careful evaluation, stabilization, and timely intervention. Efficient trauma systems and rapid assessment protocols are vital to minimize delays in presentation, factors known to influence outcomes in pelvic fractures. The correct timing of surgical intervention has been controversial. Some studies provide evidence to intervene within 8-72 hours as soon as resuscitation has been achieved [19-21], while others propose delayed intervention to minimize the second-hit phenomenon and blood loss [22]. In our resource-constrained region, delays in patient care are exacerbated by the scarcity of fellowship-trained pelvic surgeons. The absence of specialized expertise prolongs the diagnostic and treatment phases for pelvic fractures. Additionally, delayed referrals and extended travel times from other provinces contribute to a protracted timeline in accessing timely and specialized interventions, underscoring the urgent need for enhanced regional collaboration and capacity building in trauma care.

In terms of intraoperative parameters, the median intraoperative blood loss of 250 ml is relatively low, emphasizing the importance of meticulous surgical techniques in minimizing blood loss during pelvic fracture fixation. This can be due to the fact where Iliosacral screws were placed percutaneously utilizing a stab incision as compared to conventional open approaches for acetabulum reconstruction. Postoperative care, as indicated by ICU and SCU stays, plays a pivotal role in the recovery process. Notably, our findings highlight the need for specialized care, with a significant association between complications and the length of stay in specialized care units. This emphasizes the importance of individualized patient management strategies using a multi-disciplinary approach and continuous monitoring in the postoperative period [23].

The study's strengths include its prospective design, use of a diverse patient cohort, and comprehensive examination of variables affecting morbidity in pelvic fracture patients. Focusing on 30-day postoperative outcomes provides critical insights into immediate complications, thereby informing clinical decision-making and contributing to improved patient care in the context of a tertiary care setting in Karachi, Pakistan. The limitations of our study include its single-center nature and the relatively small sample size. Additionally, the short-term focus may not capture long-term complications or outcomes. Future research should explore larger cohorts and consider long-term follow-up to provide a more comprehensive understanding of pelvic fracture outcomes.

## Conclusions

Our study adds valuable insights into the short-term morbidity and mortality outcomes following pelvic fracture fixation in a tertiary care hospital in Karachi, Pakistan. By examining a diverse set of parameters and complications, we aim to contribute to the existing knowledge base and guide clinical decision-making. The findings underscore the need for a nuanced, multidisciplinary approach to managing pelvic fractures to minimize complications and improve patient outcomes. Ultimately, our research seeks to bridge critical gaps in understanding and directly impact the clinical management of pelvic fracture patients at our tertiary care center.
